# Correction to “Multimodal Treatment of a Peripheral Primitive Neuroectodermal Tumor Originating From the Thoracic Cavity in a Dog”

**DOI:** 10.1111/jvim.70130

**Published:** 2025-05-19

L. H. Ackerman, M. Toonder, S. Bosch, V. B. Semenova, T. P. Spicer, O. Alas, L. S. Thorsen, A. G. Menoyo, V. B. Stevenson, J. R. Gutti, P. V. Saavedra, R. Nance, B. Sahay, G. P. Hery, A. M. Chan, M. E. Salute, N. Bensilmane, V. F. Vega, and R. J. Milner, “Multimodal Treatment of a Peripheral Primitive Neuroectodermal Tumor Originating From the Thoracic Cavity in a Dog,” *Journal of Veterinary Internal Medicine* 39, no. 2 (2025): e70050. https://doi.org/10.1111/jvim.70050.

In the above mentioned article, the name Bakash Sahay has been corrected to Bikash Sahay, and Nesrine Bensilmane has been corrected to Nesrine Benslimane.

We apologize for this error.

